# Profile of patients seen at food allergy outpatient unit in the municipality of taubaté

**DOI:** 10.1186/1939-4551-8-S1-A132

**Published:** 2015-04-08

**Authors:** Ana Carolina Da Matta Ain, Anna / Paula Carelli, Marriele Moraes, Ciro Bertolli

**Affiliations:** 1 Universidade De Taubaté – Taubaté (SP), Brazil

## Background

Due to an increase both in the diagnosis of cow’s milk protein allergy in our municipality and, as a result, in the demand for the use of free amino acid formulas, the need for creating a food allergy outpatient department coordinated by an allergist and a pediatric gastroenterologist has arisen in order to see infants referred from Taubaté Healthcare Center.

## Methods

A retrospective study was conducted by analyzing medical charts of patients seen at food allergy outpatient unit from May 2013 to May 2014.

## Results

In such period, 100 new patients were seen, mainly comprised of male (64%). Most patients (69%) were under 6 months old at the time of their first visit. In 41% of the patients, the first contact with cow’s milk protein occurred before 1 month old and only 21% had such contact after 6 months of age. One (1%) patient had symptoms with no direct exposure to cow’s milk (allergy linked to breast milk). Respiratory symptoms, whether alone or associated with other complaints, were the most frequent ones in 81% of the cases (rhinitis 32%), followed by dermatitis in 44% and vomiting in 20%. Patients assessed at first visit were on cow’s milk (LV) in 30%, soy milk 30%, breast milk (LM) 9%, partially hydrolyzed (PH) 11%, extensively hydrolyzed (EH) 9.5%, free amino acids (AA) 9.5% and 1 patient on calcium replacement. Following first evaluation, they were switched to: soy 35%, EH 30%, PH 22%, LV 6.5%, AA 6.5%, and LM 1%. At the time of first visit, 23.5% of patients on cow’s milk protein haven’t started treatment yet or were partially treated, thus requiring switch to another formula. Soy was properly indicated in 30% of patients, with no need for changing formula. In service experience, PH formula showed good results in patients whose relatives were atopic and/or oligosymptomatic. EH formulas were the most indicated ones (30%) at this first evaluation; therefore decreasing AA prescriptions (5.5%) which were below the numbers described in literature.

## Conclusions

We can conclude from this data that despite current encouragement of breastfeeding, the high index of early weaning before 30 days old is a significant finding in our study of cases. Another important aspect was the high index of untreated or inadequately treated patients, stressing the importance of a specialized service in reducing food allergy-related comorbidities, thus improving patient’s quality of life and lowering costs to State.

